# GABAergic Neurotransmission in Human Tissues Is Modulated by Cannabidiol

**DOI:** 10.3390/life12122042

**Published:** 2022-12-06

**Authors:** Gabriele Ruffolo, Alessandro Gaeta, Beatrice Cannata, Camilla Pinzaglia, Eleonora Aronica, Alessandra Morano, Pierangelo Cifelli, Eleonora Palma

**Affiliations:** 1 Department of Physiology and Pharmacology, Istituto Pasteur-Fondazione Cenci Bolognetti, University of Rome Sapienza, 00185 Rome, Italy; 2 IRCCS San Raffaele Roma, 00163 Rome, Italy; 3 Department of (Neuro)Pathology Amsterdam Neuroscience, Amsterdam UMC Location University of Amsterdam, Meibergdreef 9, 1105 AZ Amsterdam, The Netherlands; 4 Stichting Epilepsie Instellingen Nederland, 0397 Heemstede, The Netherlands; 5 Department of Human Neuroscience, University of Rome Sapienza, 00185 Rome, Italy; 6 Department of Applied Clinical and Biotechnological Sciences, University of L’Aquila, 67100 L’Aquila, Italy

**Keywords:** GABA_A_ receptor, neurophysiology, epilepsy

## Abstract

Recently, the potential use of phytocannabinoids (pCBs) to treat different pathological conditions has attracted great attention in the scientific community. Among the different pCBs, cannabidiol (CBD) has showed interesting biological properties, making it a promising molecule with a high security profile that has been approved for treatment as an add-on therapy in patients afflicted by severe pharmaco-resistant epilepsy, including Dravet syndrome (DS), Lennox–Gastaut syndrome (LGS) and tuberous sclerosis complex (TSC). CBD is pharmacologically considered a “dirty drug”, since it has the capacity to bind different targets and to activate several cellular pathways. GABAergic impairment is one of the key processes during the epileptogenesis period able to induce a generalized hyperexcitability of the central nervous system (CNS), leading to epileptic seizures. Here, by using the microtransplantation of human brain membranes approach in *Xenopus* oocytes and electrophysiological recordings, we confirm the ability of CBD to modulate GABAergic neurotransmission in human cerebral tissues obtained from patients afflicted by different forms of pharmaco-resistant epilepsies, such as DS, TSC, focal cortical dysplasia (FCD) type IIb and temporal lobe epilepsy (TLE). Furthermore, using cDNAs encoding for human GABA_A_ receptor subunits, we found that α1β2 receptors are still affected by CBD, while classical benzodiazepine lost its efficacy as expected.

## 1. Introduction

Cannabis *sativa* has been used for centuries for the treatment of different pathological conditions [1]. Among the hundreds of compounds present in cannabis flowers, over 100 phytocannabinoids (pCBs) have been discovered [1]. pCBs are lipidic molecules synthetized as acid compounds and then decarboxylated when dried or exposed to heat [1]. Among pCBs, CBD and Δ9-tetrahydrocannabinol (Δ9-THC) are the most studied molecules. They share a common precursor, namely, cannabigerol (CBG) [2]. After the discovery of Δ9-THC [3], new receptors were cloned, namely, cannabinoid receptors type 1 (CB1Rs) and type 2 (CB2Rs) [4,5]. In detail, CB1Rs are G-coupled metabotropic receptors that are highly expressed in the central nervous system (CNS), and are particularly abundant in the hippocampus, the basal ganglia and the cerebellum. They are located pre-synaptically on both the excitatory and inhibitory terminals, and they are activated mainly by Δ9-THC, responsible for the psychoactive effect of this compound. On the other hand, CB2Rs are mainly expressed on immune cells and in the peripheral nervous system [1].

However, up to now, other receptor targets for pCBs have been discovered, such as the transient receptor potential vanilloid type 1 (TRPV1) [6]. Indeed, it has been demonstrated that CBD can directly modulate these receptors and is able to inhibit the enzymatic inactivation of the main endocannabinoid anandamide (AEA). CBD is also able to modulate opioid receptors binding to an allosteric site of the µ and δ opioid receptors, adding further support to its anti-nociceptive action [7]. Furthermore, pCBs can interact with the G protein-coupled receptor GPR55 involved in glutamate release regulation [8,9] and voltage-gated calcium channels (VGCCs), thus regulating CNS excitability [10,11]. Moreover, other studies have highlighted the capability of pCBs to modulate glycine receptors [12,13], serotonin receptors [14,15] and acetylcholine receptors [16], opening new perspectives for their potential use in different pathological conditions.

Lately, it was demonstrated that pCBs, and CBD in particular, were able to significantly affect CNS excitability by acting on GABAergic neurotransmission at both the pre- and post-synaptic level [17]. In addition, CBD was recently approved by the Food and Drug Administration (FDA) and the European Medicines Agency (EMA) as an anti-seizure medication for three different neuropathologies characterized by pharmaco-resistant spontaneous recurrent seizures (SRSs), namely, Dravet syndrome (DS), Lennox-Gastaut syndrome (LGS) and tuberous sclerosis complex (TSC) [18,19,20].

However, studies showing CBD’s effects on GABA_A_ receptors (GABA_A_Rs) from human epileptic tissues are not fully elucidated for two different reasons: the difficulty to obtain a proper amount of human material to perform electrophysiological recordings and, even more importantly, the lack of non-epileptic human tissues to compare the effects of CBD in normal versus pathological conditions.

In this study, taking advantage of the technique of voltage clamp recordings in *Xenopus* oocytes microinjected with human tissues [21], we bypass most of these problems, since we are able to record GABAergic currents from surgical and post-mortem tissues of human pharmaco-resistant epilepsies: DS, TSC, focal cortical dysplasia (FCD) type IIb and temporal lobe epilepsy (TLE). In addition, this approach is very powerful when human tissue is too scarce because of the rarity of these epileptic diseases, since it is possible to record neurotransmitter-evoked currents from just a few milligrams of brain tissue. Moreover, with this technique, we were able, as mentioned, to compare the CBD-mediated effect on evoked GABA currents (I_GABA_) in control healthy brain samples [22] versus epileptic brain tissues.

## 2. Materials and Methods

### 2.1. Patients

All the patients’ tissues (Table 1) used to perform the experiments reported here have been selected from the databases of the Department of Neuropathology, Amsterdam University Medical Center, University of Amsterdam (Amsterdam, The Netherlands); the Department of Neuropathology, University Medical Center Utrecht (Utrecht, The Netherlands); and the National Institute of Child Health and Human Development Brain and Tissue Bank for Developmental Disorders (Baltimore, MD, USA).

The patients in Table 1 underwent neurosurgical intervention for the treatment of pharmaco-resistant epilepsy due to TLE or FCDIIb. After resection, the tissue samples were instantaneously snap-frozen in liquid nitrogen, processed and subsequently used to perform our electrophysiology experiments. All the autopsies from which we obtained the DS and TSC samples were performed no more than 24 h after death, upon obtainment of specific written consent for the subsequent use for research purposes. Control cases (two samples: 61 and 38 years old, males) did not have a prior history of epilepsy, possessed a normal cortical histology that matched their age and did not have any relevant neuropathology. Both controls died of myocardial infarction. As previously reported, we already analyzed the immunoreactivity profiles of cortical tissues from autopsies and surgeries and found only slight differences between these [23,24]. All the procedures which required the use of human material were carried out following the guidelines from the Declaration of Helsinki and the Amsterdam UMC Research Code provided by the Medical Ethics Committee.

### 2.2. Membrane Preparation

The tissues were shipped in dry ice and either directly prepared for the electrophysiological recordings or conserved at −80 °C. The protocols for human membrane extraction and their microinjection in *Xenopus* oocytes have already been published and these procedures were carried out as previously described [25]. Concisely, tissues were homogenized in a membrane buffer solution (in mM: glycine 200, NaCl 150, EGTA 50, EDTA 50 and sucrose 300; plus 20 µL of protease inhibitors (P2714, Sigma); pH 9, adjusted with NaOH). Then, the samples were centrifuged for 15 min at 9500× *g*. Subsequently, the supernatant was centrifuged for 2 h at 100,000× *g* with an ultra-centrifuge (Beckman-Coulter). The pellet was rinsed with sterilized water and re-suspended in assay buffer (glycine 5 mM) for immediate use or stored at −80°. The use of laboratory animals (*Xenopus laevis)* and all the related procedures (surgery, oocytes extraction and their utilization) were validated by the Italian Ministry of Health and followed its guidelines (authorization no 427/2020-PR). 

### 2.3. Xenopus oocytes Electrophysiology

The electrophysiological recordings on *Xenopus* oocytes were performed 24–48 h after cytoplasmic injection [25] using the technical approach of two-electrode voltage clamp. We recorded GABA-evoked currents (I_GABA_) [26] after placing the oocytes in a 0.1 mL recording chamber and continuously perfusing them with oocyte Ringer solution (OR, in mM: NaCl 82.5; KCl 2.5; CaCl_2_ 2.5; MgCl_2_ 1; Hepes 5, adjusted to pH 7.4 with NaOH) at room temperature (20–22 °C). The perfusion system was operated by a computer connected to a gravity-driven multi-valve device (9–10 mL/min; Biologique RSC-200; Claix, France), which granted precise control of the duration of GABA applications. With this setup, the whole volume of the recording chamber was entirely replaced in 0.5 to 1 s. For all the microtransplanted oocytes, we tested the stability of I_GABA_ by assessing two consecutive GABA pulses, separated by a 4 min wash-out. In order to evaluate the acute effect of CBD (2 μM for 10 s), we used only the cells characterized by a <5% I_GABA_ modification. We defined I_GABA_ variation as a percent increase or decrease in the mean current elicited by two GABA applications before and after exposure to CBD. The GABA current run-down was defined as the decrease in the peak current amplitude after six consecutive GABA applications (10 s) spaced out by 40 s of wash-out and expressed as a percentage (I_6th_ peak/I_1st_ peak × 100). In another set of experiments, we used human GABA_A_R subunits encoding cDNAs (pCDM8 vector α1β2γ2 ratio 1:1:2; α1β2 ratio 1:1; cDNAs were a kind gift from Dr. K. Wafford). In this case, we performed an intranuclear injection in *Xenopus* oocytes with a pressure microinjector (PLI-100, Warner Instruments, Holliston, MA, USA).

### 2.4. Statistics

We assessed normal distribution with the Shapiro–Wilk test in order to choose parametric (Student’s t-test) or non-parametric (Wilcoxon signed rank test, Mann–Whitney rank sum test) tests before starting the data analysis process. Data were statistically analyzed using Sigmaplot 12 software, and differences between two data sets were considered significant when *p* < 0.05. Oocytes used in each experiment are indicated as (n). 

## 3. Results

- **CBD modulation on GABA-evoked currents in *Xenopus* oocytes micro-transplanted with DS human tissues**

**Figure 1:**
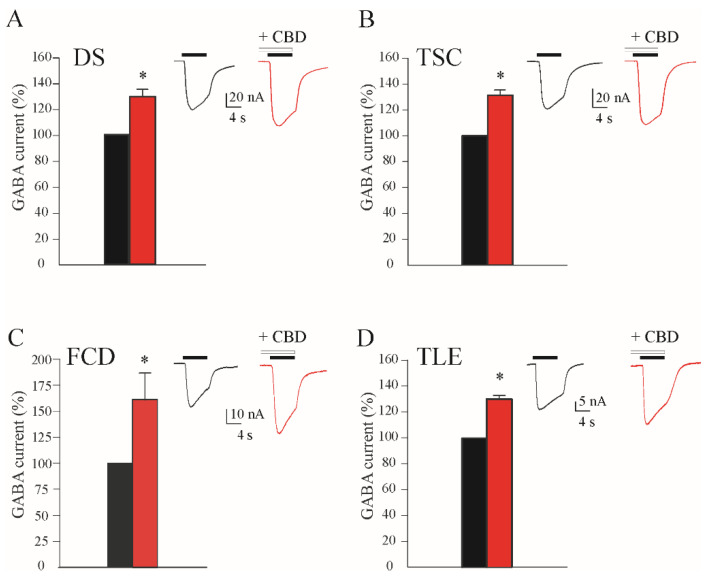
CBD effect on GABA currents (I_GABA_) amplitude in oocytes transplanted with tissues from (**A**) Dravet syndrome (DS, I_GABA_ range: from 11.2 to 122.4), (**B**) tuberous sclerosis complex (TSC, I_GABA_ range: from 7.3 to 85.1), (**C**) focal cortical dysplasia type IIb (FCD, I_GABA_ range: from 6.8 to 72.3) and (**D**) temporal lobe epilepsy (TLE, I_GABA_ range: from 6.2 to 89.3) patients. All the bar-graphs show the % variation in the mean current amplitude after incubation with CBD 2 μM (black, % current before incubation; red, % current after incubation), * *p* < 0.05. In each panel, the *inset* represents sample currents with or without CBD. Black horizontal bars represent GABA 50 μM application; white horizontal bars represent CBD 2 μM application.

DS is an epileptic disorder characterized by pharmaco-resistant recurrent seizures. CBD showed promising results both in animal models and in clinical trials [27,28,29], and was, thus, approved to treat this condition both in the USA and Europe [18,19]. In this set of experiments, we were able to test the effect of an acute application of CBD in two brain samples obtained from adult DS patients (Table 1). After the injection of oocytes with this membrane preparation, we elicited GABA-evoked currents (I_GABA_) with applications of 4 s of GABA (50 μM) (mean −65.0 ± 7.1 nA; Figure 1A; n = 20; #1, 2, Table 1). 

In order to confirm the recordings of genuine I_GABA_, we completely blocked these currents with high concentrations of bicuculline (100 μM; not shown). For comparison, we recorded, with the same approach, I_GABA_ from oocytes injected with membranes obtained from two control patients without any neurological disease (see Methods). We found a comparable I_GABA_ amplitude (mean −63.3 ± 3.5 nA; n = 12). When we recorded I_GABA_ after the acute co-application of GABA and CBD, we found a significant increase in the elicited I_GABA_ (+27.4 ± 4.8%; GABA 50 μM; CBD 2 μM; Figure 1A; n = 16; *p* < 0.05; #1–2; Table 1). This CBD effect on I_GABA_ was fast and reversible, since after 5 min of washing with the oocyte Ringer solution (OR), the I_GABA_ amplitude recovered to pre-treatment values (−2.7% ± 5.7%; GABA 50 μM; n = 8; *p* > 0.05 compared to the control current; #1–2; Table 1). Afterwards, with the same approach, we tested the effect of CBD on the oocytes injected with the control cortical membranes obtained from the autopsies of healthy patients. Interestingly, we found that CBD was able to increase the amplitudes of I_GABA_ in the control tissues (+25.5 ± 2.4%; GABA 50 μM, n = 10; *p* < 0.05, CBD 2 μM), similarly to what was showed in DS.

- **CBD modulation on GABA-evoked currents in *Xenopus* oocytes micro-transplanted with TSC human tissues**

Since the use of CBD was approved by both the FDA and the EMA to treat TSC patients as an add-on therapy [20], we obtained surgical brain samples from these patients in order to evaluate if CBD was able to modulate I_GABA_ in this genetic epileptic condition. Indeed, TSC is a genetic, rare and multi-systemic disease characterized by different neurological alterations, including strong pharmaco-resistant seizures [30]. Different studies have highlighted an altered neurotransmission in this pathology, engaging both an inhibitory and excitatory transmission [31]. CBD was able to significantly increase evoked I_GABA_ when applied together with GABA (CBD = +29.4 ± 3.0%; GABA 50 μM; CBD 2 μM; n = 18; *p* < 0.05; #3 and #4; Table 1; Figure 1B). Again, also in this case, the effect was reversible, since 5 min of wash-out with OR was able to recover the I_GABA_ amplitudes to pre-CBD levels (−4.0% ± 2.5%; GABA 50 μM; n = 10; *p* > 0.05 compared to the control current; #1–2; Table 1).

- **CBD modulation on GABA-evoked currents in *Xenopus* oocytes micro-transplanted with FCDIIb human tissues**

FCDs are a group of pathological conditions characterized by an altered cortical development often associated with pharmaco-resistant epilepsy [32,33,34]. Among them, FCD type IIb represents the most common malformation of cortical development [34]. The histopathological hallmark of FCDIIb is represented by an altered cortical lamination and the presence of cellular abnormalities (e.g., balloon cells and dysmorphic neurons) linked to an altered inhibitory neurotransmission, leading thus to SRSs [35,36]. We investigated the possible effect of CBD on GABA_A_Rs using brain tissues from FCD patients that underwent epilepsy surgery. We used the same approach used above for DS and TSC, recording bicuculline-sensitive I_GABA_ (not shown) in order to test the CBD effect on these human samples. We obtained a statistically significant increase in I_GABA_ amplitudes (CBD = +60.5 ± 25.5%; GABA 50 μM; CBD 2 μM; n = 11; *p* < 0.05; #8 and #9; Table 1; Figure 1C) that was, as in the case of DS and TSC, completely washable after 5 min of wash-out with OR solution (not shown).

- **CBD modulation on GABA-evoked currents in *Xenopus* oocytes micro-transplanted with TLE human tissues**

**Figure 2:**
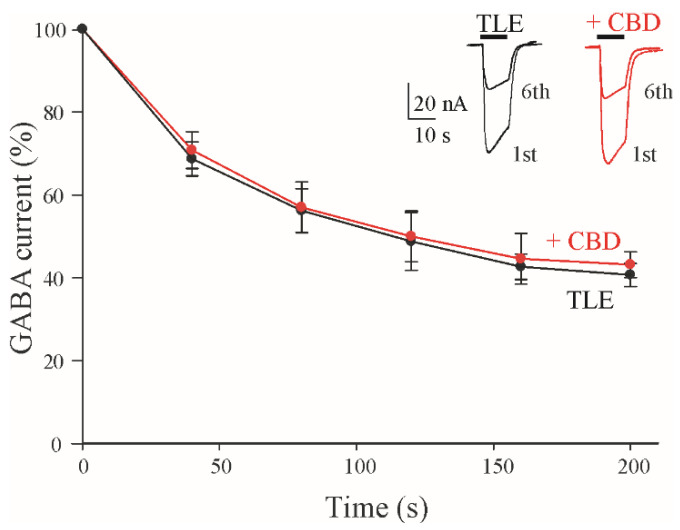
GABA current run-down in oocytes microtransplanted with mTLE tissues. Time course of GABA current run-down relative in mTLE without (●) and with (CBD) (●) application. I_GABA_ were normalized to those elicited by the first GABA application. Data are expressed as mean ± SEM. *Inset* represents superimposed first and sixth GABA applications (500 μM) without (black traces) or with CBD (red traces). Black horizontal bars represent GABA application; CBD 2 μM was perfused during the run-down protocol (in red).

TLE represents the most common type of focal epilepsy in adulthood [37], and based on seizure semiology, it can fall into two different categories: the most common mesial form, which is characterized by mesial temporal lobe (mTLE) symptoms, and a rarer form with lateral temporal lobe symptoms (lTLE) [38]. Usually, after an epileptogenic insult (i.e., head trauma, stroke, brain tumor, brain infection), several pathological and physio-pathological alterations occur, and after a latent period that can last from hours to years, the brain becomes epileptic [37]. One of the main alterations that occurs during epileptogenesis, responsible for the recurrent seizures, is an imbalance between the excitatory and inhibitory neurotransmissions [39]. In particular, an increased use-dependent desensitization (i.e., GABA current run-down) of the GABA_A_Rs was described as a pathological hallmark of pharmaco-resistant mTLE [40,41,42]. Briefly, GABA_A_Rs from mTLE tissue become less responsive to repeated activation than those from healthy control tissue [41]. This GABA current run-down may imply hyper-excitability. In our experiments, we found that CBD application (2 μM) was able to induce a washable increase in I_GABA_ (CBD = +30.4 ± 2.2%; GABA 50 μM; n = 20; #5–7; Table 1; Figure 1D). In another set of experiments, we tested whether CBD was able to improve the GABA_A_ current run-down in order to restore a more physiological GABAergic neurotransmission. As previously reported [42,43], the application of 500 μM GABA in the oocytes injected with the membranes from the cortex of mTLE patients exhibited a statistically significant current run-down (the I_GABA_ elicited by the sixth GABA application fell to 40.7 ± 2.8 %; n = 12; #5–7; Table 1; Figure 2). 

Surprisingly, an acute application of CBD 2 μM did not significantly change the rate of run-down (42.9 ± 2.8%; GABA+ CBD 2 μM; n = 16; #5–7; Table 1), while the increase in I_GABA_ currents still persisted, even if to a lesser extent. This last evidence suggests that the CBD effect on GABA current amplitudes is not linked to the desensitization processes.

- **CBD modulation of GABAergic neurotransmission of α1β2 GABA_A_ receptors**

**Figure 3:**
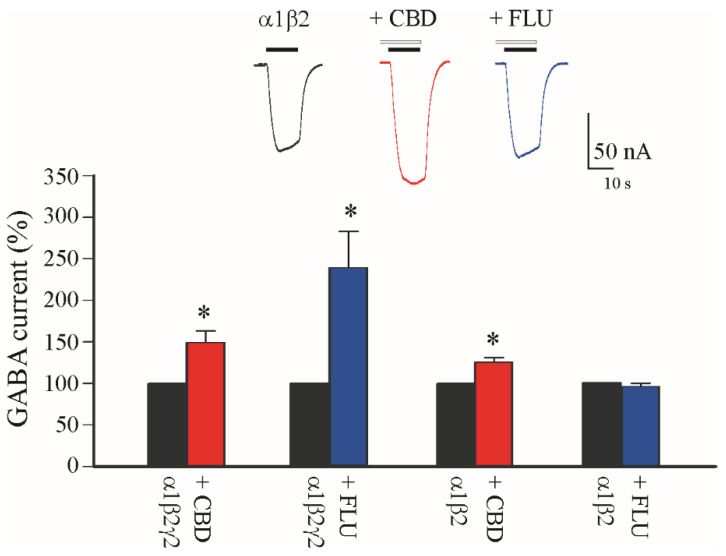
CBD effect in oocytes expressing α1β2γ2 and α1β2 GABA_A_ receptor subunits. Bar graphs show the GABA current increase (as %) induced by CBD 2 μM (red bars) and flunitrazepam (FLU, blue bars) 6 μM in oocytes intranuclearly injected with cDNAs encoding human α1β2γ2 and α1β2 GABA_A_R subunits, * *p* < 0.05. In the *inset*, representative current traces of the experiment with α1β2-containing GABA_A_Rs in control condition (black trace), and with CBD (red trace) and FLU (blue trace) as indicated; note the ineffectiveness of FLU in this condition. Black horizontal bars represent GABA 5 μM application, white horizontal bars represent CBD 2 μM application.

To better understand the CBD effect on I_GABA_ amplitudes, we performed another set of experiments on oocytes expressing single GABA_A_Rs without ancillary or associated proteins. Thus, we intranuclearly injected cDNAs encoding for the most common GABA_A_R subunits in CNS (α1β2γ2) with or without the γ GABA_A_R subunit that is involved in the benzodiazepine (BDZ) binding site [44]. In these conditions, CBD was able to significantly increase the I_GABA_ amplitudes (α1β2γ2: +48.0 ± 13.0%; GABA 5 μM; Figure 3; n = 6). For comparison, we also tested BDZ flunitrazepam (FLU, 2 μM), and we found, confirming previous reports [45], a strong potentiation of the elicited I_GABA_ (+140 ± 43%; GABA 5 μM; Figure 3; n = 6). Subsequently, we expressed α1β2-containing GABA_A_Rs, and we found that, while the application with FLU was completely ineffective in increasing I_GABA_ (−4 ± 3.5%; GABA 5 μM; Figure 3; n = 6), when we applied CBD, we still found a significant increase in I_GABA_ (α1β2: +24.5 ± 3.8%; GABA 5 μM; Figure 3; n = 6).

## 4. Discussion

In this paper, using human epileptic brain tissues, we were able to highlight a modulation of CBD on the function of GABA_A_Rs. GABA impairment is a well-established mechanism involved in brain hyperexcitability [46]. Indeed, several studies have demonstrated how GABA_A_R subunits’ composition and functional properties change in epileptic disorders [42,47]. Furthermore, some of the most used ASMs, such as BDZ and barbiturates, act specifically on this class of ionotropic receptors, since GABA_A_R represents one of the main pharmacological targets in epileptic disorders, and the recovery of its physiological function could lead to a correct inhibitory neurotransmission [46]. In addition to BDZ and other GABAergic ASMs, pCBs represent a new class of pharmacological tools that show promising results in pre-clinical and clinical studies. Specifically, CBD obtained approval for use in some strongly pharmaco-resistant epilepsies such as DS, LG syndrome and, lately, TSC, as an add-on therapy [18,19,29]. The mechanism by which CBD and other pCBs exert their therapeutic effects still remains partially unclear, as multiple different targets have been identified [6,7,8,9,10,12,13,14,15,16,48]. Here, taking advantage of the micro-transplantation of human brain membranes in *Xenopus laevis* oocytes, we confirm that CBD can affect GABAergic neurotransmission by acting on human GABA_A_Rs. We tested CBD on four different epileptic conditions (DS, TSC, FCDIIb and mTLE), all characterized by a strong pharmaco-resistance to canonical ASMs, showing that CBD can significantly and reversibly potentiate GABAergic-evoked currents in all these diseases. Interestingly, in FCDIIb, the CBD effect seems more significant, thus suggesting that this potentiation may also be related to different cellular subtypes and/or different GABA_A_R arrangements. Further studies using human slices from FCDIIb patients could better elucidate this specific point. However, we can hypothesize that CBD’s ability to reduce the frequency and severity of epileptic seizures [18,19] is, at least in part, due to an interaction with GABA_A_Rs. In addition, we demonstrated that CBD also carries out its action on control tissues, indicating that this compound can also modulate the normal function of GABA_A_Rs. Interestingly, the aforementioned CBD effect is quite fast and rapidly washable, suggesting that its action is not mediated by the activation of intracellular pathways, as previously shown for other ASMs [46,49]. In line with this evidence, we did not observe any CBD effect on GABA current run-down in mTLE, an impairment that is prevented by acting on the phosphorylation mechanisms of GABA_A_Rs and/or their associated proteins [23,40,42]. To further exclude the possibility that CBD could interact with endogenous proteins and/or the activation of host cell intracellular signaling, we expressed, via the intranuclear injection of cDNAs, human GABA_A_Rs formed by α1β2γ2, the most common subunit composition, showing again a clear increase of I_GABA_. Altogether, these results strengthen our hypothesis that CBD can directly bind to GABA_A_Rs, increasing its efficacy, and that this effect is not mediated by specific CBRs [2,50]. Moreover, in different forms of epilepsy, GABA_A_R undergoes different rearrangements of its subunit composition, such as in DS [45] or mTLE [42,47]. These subunit changes can modify GABA_A_R’s function, often determining a reduced inhibitory tone and altering the effectiveness of ASMs on the original molecular target [51]. This hypothesis is strengthened by several studies that clearly showed CBD’s ability to modulate different GABA_A_R subunit compositions [17,45], such as α2, α3 and α6, indicating, thus, that its action is not linked to the different α subunit expressions. In the aforementioned conditions, drugs targeting new and alternative modulatory sites on GABA_A_Rs are likely to yield better results compared to classical ASMs. Noteworthily, we showed that CBD’s effect on I_GABA_ still persists in α1β2—GABA_A_Rs, while a classical BDZ [44] did not show any significant effect. This last finding suggests that CBD may act also on defective GABA_A_Rs, especially in those conditions where BDZs are partially or completely ineffective [52]. Interestingly, as demonstrated in other studies [17], CBD is also able to modulate δ-containing GABA_A_Rs, making it an interesting pharmacological tool to potentially treat other epileptic pharmaco-resistant conditions characterized by a dysfunction of “tonic” GABA_A_R neurotransmission [53]. Further studies are required in order to better characterize CBD’s modulation of GABA_A_R’s function and identify the exact site of CBD binding on human GABA_A_Rs. In conclusion, we can hypothesize that CBD, being devoid of relevant psychotropic effects [11,54], can open new perspectives for its use, not only in epileptic diseases, but also in the treatment of other neurological and psychiatric conditions where GABAergic transmission is impaired, such as attention-deficit hyperactivity disorders (ADHD) [55] and neurodegenerative diseases [56,57,58]. 

## Figures and Tables

**Table 1:** Patients’ clinical data.

Patient	Age	Gender	Duration of Epilepsy	Brain Region	Type of Seizures	Diagnosis/Mut/Cause of Death	ASMs
**#1**	49	M	48	T	FIAS/GS	DS/SCN1A mut/heart failure	CLB, STP, VPA
**#2**	46	F	44	T	FIAS/GS	DS/SCN1A mut/ bronchopneumonia	CLB, STP, VPA
**#3**	47	M	35	T	FAS	TSC/TSC2 mut/Myocardial infarction	PHB, VPA, CBZ, CLB
**#4**	42	F	41	T	FIAS	TSC/TSC2 mut/Myocardial infarction	PHB, VPA, CBZ
**#5**	41	M	21	T	FIAS/GS	TLE-HS	CBZ, TPM
**#6**	54	F	42	T	FIAS/GS	TLE-HS	CBZ, LMT, PHB
**#7**	52	M	42	T	FIAS/GS	TLE-HS	CBZ, PHB, VGB
**#8**	18	M	16	T	FAS	FCDIIb/mTOR mut	CBZ, VPA, LMT, LCM
**#9**	45	M	34	T	FAS	FCDIIb	LEV, OCZ

Patients #1, 2: tissues from autopsies of patients affected by Dravet syndrome (DS) with SCN1A mutation; Patients #3 and 4: tissues from tuberous sclerosis complex (TSC) patients (TSC2 mutation). All autopsies were performed within 24 h of death. Patients #5–7: temporal lobe epilepsy (TLE) patients with hippocampal sclerosis (HS). Patients #8 and 9: tissues from focal cortical dysplasia type IIb (FCDIIb). ASMs—anti-seizure medications; CBZ—carbamazepine; CLB—clobazam; F—female; FAS—focal aware seizures; FIAS—focal impaired awareness seizures; GS—generalized seizures; LCM—lacosamide; LEV—levetiracetam; LMT—lamotrigine; M—male; mut—mutation; OCZ—oxcarbazepine; PHB—phenobarbital; STP—stiripentol; T—temporal; TPM—topiramate; VGB—vigabatrin; VPA—valproic acid.

## Data Availability

Not applicable.
